# A Randomized, Multicenter, Double-Blind, Placebo-Controlled Clinical Trial to Assess the Efficacy and Safety of a Polyherbal Formulation in Men With Erectile Dysfunction

**DOI:** 10.7759/cureus.79613

**Published:** 2025-02-25

**Authors:** Gayatri P Ganu, Pramod K Kolsure, Sanman S Kolhe, Aditya Dev, Somesh S Shintre, Shrikant G Dhavale, Vinod K Kuber, Dheeraj H Nagore, Sriram Padmanabhan

**Affiliations:** 1 Department of Pharmacology, Mprex Healthcare Pvt. Ltd., Pune, IND; 2 Department of Formulation and Development, SAVA Healthcare Limited, Pune, IND; 3 Department of Research and Development, SAVA Healthcare Limited, Pune, IND; 4 Department of Biochemistry, SAVA Healthcare Limited, Pune, IND

**Keywords:** erectile dysfunction, pde-5, polyherbal formulation, sexual function, testosterone levels

## Abstract

Introduction

Erectile dysfunction (ED) is a prevalent condition impacting men worldwide, often causing significant reductions in quality of life, emotional well-being, and relationship satisfaction. ED currently affects approximately 100 million men worldwide, with its prevalence projected to rise to 320 million by 2025, particularly in developing nations. Notably, nearly half of all men aged 40 and above experience some degree of ED, underscoring its widespread impact on male health. Existing treatments, while available, frequently fall short in terms of efficacy and safety, underscoring the demand for innovative and safer therapeutic alternatives.

Methods

Eighty-five participants completed the study and were randomly assigned to receive either the test treatment (polyherbal formulation) or a placebo, utilizing a computer-generated randomization list. The study protocol included screening (up to 14 days prior), baseline assessment (Day 0), and follow-up visits at day 30, day 60, and day 90 (± 5 days), allowing for thorough monitoring of outcomes.

Results

The test group exhibited significantly greater improvements in erectile function (92.2% vs. 34.8%), along with enhancements in sexual desire, orgasmic function, intercourse satisfaction, and overall satisfaction compared to the placebo group. Quality of erection scores also showed marked improvement in the test group. Additionally, serum testosterone levels increased by 46.5% in the test group compared to a mere 3.3% in the placebo group. Adverse events were mild and occurred with similar frequency in both groups (9.3% vs. 9.5%). Overall, the test group demonstrated robust efficacy and a favorable safety profile, presenting a promising therapeutic option for the management of ED.

Conclusion

The in-vitro phosphodiesterase type 5 (PDE5) assay and clinical study demonstrate the effectiveness of test intervention in managing ED and improving sexual health, with a favorable safety profile. Its synergistic herbal ingredients enhance efficacy, making it a promising solution in the management of ED.

## Introduction

Erectile dysfunction (ED) is not just a physical health issue, it profoundly affects a man’s emotional and psychological well-being, often leading to anxiety, stress, and strained relationships. Traditionally viewed as a condition affecting older men, modern research reveals an alarming rise in ED cases among men under 40 years old [1]. ED is defined as the persistent inability to achieve or maintain an erection adequate for satisfactory sexual activity [2]. ED is a prevalent medical disorder that affects broadly 100 million men globally and is now regarded as a major public health concern. It is speculated that over half of all men above the age of 40 are susceptible to ED. While in 1995, ED affected over 152 million men worldwide, it is anticipated that by 2025, more than 320 million patients will be afflicted, with the greatest expected growth in developing countries [3,4]. The prevalence of ED increases with age, as shown in the Portuguese Erectile Dysfunction Study. The total age-adjusted prevalence was 48.1%, with significant age-related differences: 29% in men aged 40-49 years, 50% in those aged 50-59 years, and 74% in men aged 60-69 years. The severity of ED also escalates with age, with complete ED reported in 1%, 2%, and 10% of men in the respective age groups [5].

There are merely two types of ED: primary and secondary. Primary ED is infrequent and is frequently associated with severe psychopathology, low testosterone levels, or genetic abnormalities. More often, secondary ED can be brought through illnesses including diabetes, arteriosclerosis, neurological disorders, psychiatric problems, protracted stress, or prior vaginal surgery. Antidepressants and blood pressure medications, in particular, can have an adverse effect, especially in elderly people [6,7].

Lifestyle modifications are the primary approach for ED management. Pharmacological options include testosterone replacement therapy (TRT), phosphodiesterase type 5 (PDE5) inhibitors, intra-cavernosal injections, vacuum constriction devices, intra-urethral prostaglandin suppositories, and penile prosthesis surgery [8]. However, these interventions can be expensive and associated with significant side effects, prompting many patients to seek alternative treatments, such as Ayurveda [9].

This growing interest in alternative therapies provides the rationale for exploring polyherbal formulation, a combination of herbal ingredients believed to target the physiological and psychological aspects of ED. *Tribulus terrestris* enhances libido and testosterone levels, while *Withania somnifera* reduces stress, a key factor in ED [10,11]. Shatavari [12] and Musali Svet promote sexual vigor and stamina [13], and Kaucha, a dopamine precursor, improves mood and sexual function [14,15]. Vidarikand rejuvenates reproductive health [16,17], Jatiphal enhances sexual desire [18], and Akarkarabha strengthens erectile function (EF) [19].

Polyherbal is hypothesized to be effective in treating ED due to the actions of its ingredients. To test this hypothesis, a clinical trial titled "Evaluation of Efficacy and Safety of Polyherbal Formulation in Participants Suffering from Erectile Dysfunction - A Randomized, Double-Blind, Placebo-Controlled, Multi-Centric, Interventional Clinical Study" was conducted. As ED increasingly affects younger men, the demand for safe, effective, and affordable treatments is growing. The study on test polyherbal formulation offers a promising avenue for men seeking alternative remedies with fewer side effects, providing hope for millions around the world looking for improved sexual health and overall well-being.

## Materials and methods

This was a randomized, double-blind (both the investigators and the study participants), placebo-controlled, comparative, interventional, multi-centric, prospective, clinical study to evaluate the efficacy of test formulation in participants suffering from ED. The study groups received either of the following treatments: The test group received a polyherbal formulation tablet and the placebo group received a placebo tablet. The duration of the treatment period was 90 days. The study was initiated only after written approval was obtained from the Independent/Institutional Ethics Committee (IEC) and subsequent registration of the study on the Clinical Trial Registry-India (CTRI) website. The study was conducted as per approved protocol and as per Good Clinical Practices guidelines given by AYUSH in March 2013. The clinical trial was registered with the CTRI under the registration number CTRI/2018/12/016697 (Registered on: 17/12/2018). Participants were enrolled in the study. The study was conducted across four centers: Jyoti Multispecialty Clinic, KVTR Ayurvedic College (Boradi), Shatayu Ayurveda and Research Center, and Sunad Ayurved. Ethical approval was obtained from two ethics committees. The IEC at Dhanashree Hospital, Navi Sangvi, Pune (ECR/19/Indt/MH/2013/RR-16) granted approval for three sites - Jyoti Multispecialty Clinic, Shatayu Ayurveda and Research Center, and Sunad Ayurved. Approval for KVTR Ayurvedic College, Boradi, was provided by the Institutional Ethics Committee, KVTR Ayurvedic College (AMB/1622/2017-2018). The clinical trial data was collected from 20/12/2018 to 13/06/2019. The composition of the test investigational product is depicted in Table 1.

**Table 1:** Investigational product composition

S. No.	Ingredients	Botanical Name	Part Used	Composition
1	Gokshur extract	Tribulus terrestris	Fruit	140 mg
2	Ashwagandha extract	Withania somnifera	Root	120 mg
3	Shatavari extract	Asparagus racemosus	Root	80 mg
4	Kaucha extract	Mucuna pruriens	Seed	55 mg
5	Musali Svet extract	Asparagus adscendens	Tuberous root	60 mg
6	Vidarikand extract	Pueraria tuberosa	Root	20 mg
7	Jatiphal extract	Myristica fragrans	Seed	20 mg
8	Akarkarabha extract	Anacyclus pyrethrum	Root	15 mg

Inclusion criteria

Male participants aged 21 to 50 years suffering from ED based on the EF domain of the International Index of Erectile Function (IIEF) score between 11 and 25 at screening visits were eligible for the study. Additionally, participants needed to be in an active and stable sexual relationship throughout the study duration. All participants were required to provide informed consent, demonstrating their willingness to participate in the clinical trial after reading and understanding the study information. Furthermore, participants had to commit to attending all required study visits.

Exclusion criteria

Participants with anatomical abnormalities of the penis, those who underwent radical prostatectomy, spinal cord injuries, or any other surgeries involving the urogenital organs were not eligible. Additionally, participants exhibiting severe sexual dysfunction characterized by an International Index of Erectile Function-Erectile Function (IIEF-EF) domain score below 11 or above 25 were excluded, particularly those who had previously failed to respond to PDE5 inhibitors or had received treatments for spermatogenic fertility within the past three months.

Participants with ED linked to primary disorders or untreated endocrine diseases, as well as those with a history of pelvic surgery, penile implants, or significant penile deformities, were also excluded. Other exclusion criteria included a history or current issues with significant alcoholism or drug abuse within the past year, recent serious health conditions within the past three months, or other major disorders at the discretion of the investigator.

Clinically significant laboratory or electrocardiogram (ECG) findings during screening, along with those receiving hormonal treatments, antidepressants, antipsychotics, or other psychoactive medications, were also excluded, encompassing hypersensitivity to any ingredients in the study drugs.

Sample size

The sample size was calculated based on the primary endpoint, which was the mean change in IIEF score of 9.0 with a standard deviation of 4.95 in the test group and a mean change of 0.61 with a standard deviation of 2.43 in the placebo group. To attain 90% power and a 5% significance level, 80 completed cases (40 in each group, i.e., in a 1:1 ratio) were required, assuming a 5% margin of error. Additional subjects were enrolled to ensure that 80 participants completed the study. Ultimately, 85 participants completed the study (test: 43, placebo: 42). Superiority clinical trial design was hypothesized and the sample size was calculated using the formula $N = \frac{2 (Z_{\alpha} + Z_{\beta})^2 (SD)^2}{(d - \epsilon)^2}$ using SPSS for Windows (Version 10.0. Chicago, SPSS Inc.) [20,21]*.*

Methodology

This is a randomized, double-blind, placebo-controlled, multi-centric, interventional, prospective clinical study of the efficacy and safety of polyherbal formulations in ED. After ethics committee approval and CTRI registration, married male participants (aged 21-50) attending the outpatient department were screened for eligibility, and informed consent was obtained. Eighty-five (85) participants completed the study, with 43 receiving a test polyherbal formulation and 42 receiving a placebo, in a 1:1 ratio. The treatment lasted 90 days, during which the efficacy of the investigational products was compared between groups. Participants were instructed to take two tablets (each 550mg) orally twice daily after meals with water.

Concomitant diseases and medications were assessed during screening. The changes in IIEF-EF, orgasmic function, intercourse satisfaction, and overall satisfaction scores were assessed. Participants maintained a daily diary card to record the number of sexual encounters and evaluated the quality of penile erection using the Quality of Erection Questionnaire (QEQ). Assessments were conducted on day 0 (baseline), day 30, day 60, and day 90 using the IIEF. Sexual desire was also assessed. Additionally, the Erectile Dysfunction Inventory of Treatment Satisfaction (EDITS) questionnaire (participant and partner versions) was administered on days 30, 60, and 90 to evaluate changes in sexual health.

**Figure 1:**
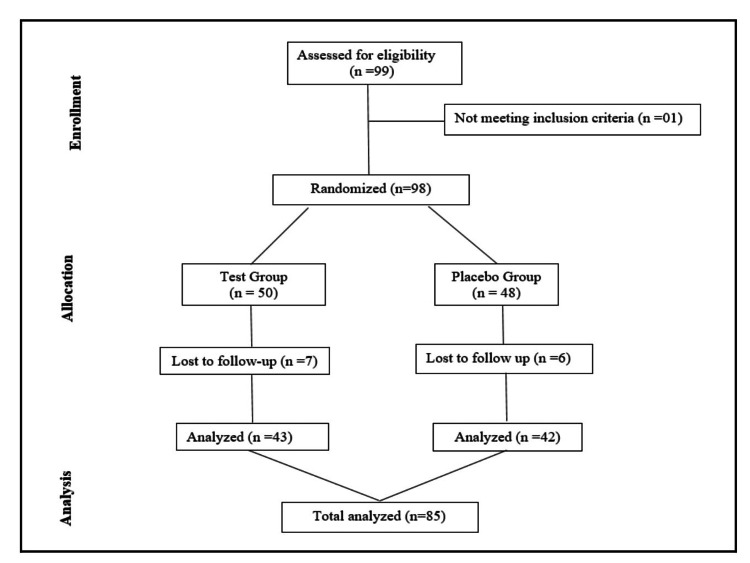
Consolidated Standards of Reporting Trials (CONSORT) diagram for the study

Laboratory assessments, including complete blood count (CBC), erythrocyte sedimentation rate (ESR), hemoglobin (Hb%), blood sugar level fasting (BSL-F), liver and renal function tests, lipid profile, urine analysis, and serum total testosterone, were conducted at baseline and day 90. Safety, including adverse events (AEs) and serious adverse events (SAEs), was monitored throughout the study. Treatment compliance and tolerability were assessed, and blood samples were collected by trained phlebotomists. The consort diagram is shown in Figure 1.

Statistical analysis

Statistical analysis was conducted using SPSS. The Kolmogorov-Smirnov (K-S) test was used to calculate the normality. Primary endpoints were evaluated with the Wilcoxon signed-rank test and comparisons between groups using analysis of variance (ANOVA) with the Kruskal-Wallis test. Secondary endpoints were analyzed using the Wilcoxon signed-rank test, Mann-Whitney U test, and ANOVA with the Kruskal-Wallis test. The tolerability of the study drug and placebo were analyzed using the Chi-square test.

Methodology of PDE5 inhibition assay

The human PDE5 enzyme (Sigma-Aldrich, St. Louis, USA, Cat #E9034) was diluted 1:100 in a buffer containing 50 mM Tris (pH 8.0) and 100 mM magnesium chloride. A fresh enzyme solution was prepared for each assay. A 10 mg/mL stock solution of the investigational formulation was prepared in water, and various concentrations of this solution were tested for their effects on PDE5A activity in vitro.

The PDE5 assay was performed using a modified protocol adapted from Lin et al. Briefly, the reaction mixture consisted of 50 mM Tris buffer (pH 8.0) and 100 mM MgCl2. To this, 7.5 μL of the PDE5 enzyme solution (from the 1:100 diluted stock) and 2.5-25 μL of the investigational formulation solution were added to assess dose-dependent inhibition. The mixture was incubated for one hour at 37°C. Following pre-incubation, 3 μM cGMP (prepared in water) was added, and the total reaction volume was adjusted to 500 μL with water. The reaction was allowed to proceed for three hours at 37°C [22].

Optimized assay conditions for screening PDE5A1 inhibitors included 0.05 μg/mL PDE5A1, 1.14 μg/mL cGMP, and a 180-minute reaction at 37°C. Post-reactions, samples were heated at 100°C for five minutes to deactivate the PDE5 enzyme. After cooling, the samples were analyzed using high-performance liquid chromatography (HPLC), and percentage inhibition was calculated based on the HPLC peak area of the remaining cyclic guanosine monophosphate (cGMP).

Chromatographic analysis

HPLC analysis was performed using a Waters HPLC system (Model: Alliance e2695, Waters Corporation, Milford, USA) equipped with Chromeleon 7 software (Thermo Fisher Scientific, Waltham, USA) and a photodiode array (PDA) detector. The mobile phase consisted of 0.05 M potassium phosphate buffer (mobile phase A) and methanol (mobile phase B). The column used was an Inertsil ODS, C18 (150 × 4.6 mm, 3.5 μm; GL Sciences Inc., Tokyo, Japan), and the detection wavelength was set at 254 nm.

The flow rate was maintained at 0.6 mL/min, with the column temperature set at 40°C, and the injection volume was 50 μL. The total run time per sample was 15 minutes. The gradient elution profile was as follows: mobile phase A: mobile phase B was maintained at a 95:5 ratio from 0.01 to 3 minutes, adjusted to 85:15 for 10 minutes, and reverted to 95:5 from 10.1 to 15 minutes. This gradient allowed the effective separation of GMP and cGMP.

## Results

Demographic details

In the test group, the mean age of participants was 36.40 ± 6.06 years, with an age range of 26 to 50 years. In the placebo group, the mean age was 35.48 ± 5.46 years, ranging from 24 to 48 years. The comparison between groups revealed no statistically significant difference (Table 2).

**Table 2:** Demographic details Data were analyzed using the Student’s t-test, and values are represented as mean ± standard deviation (SD). NS: not significant

Parameters	Test (n= 43)	Placebo (n= 42)	P-value
Age (mean ±SD)	36.40 ±6.06	35.48 ±5.46	0.464 (NS)
Range	26.00-50.00	24.00-48.00	

Assessment of improvements in sexual function between the groups

The IIEF Questionnaire assesses EF, orgasmic function, sexual desire, intercourse satisfaction, and overall satisfaction across 15 questions, each scored from 0 to 5, with higher scores indicating better sexual function. The test group showed remarkable improvements across all sexual function domains, with a 92.2% increase in EF, 98.3% in orgasmic function, 81.1% in intercourse satisfaction, and 92.1% in overall satisfaction by day 90 consistently outperforming the placebo group, which showed only modest gains (Table 3).

**Table 3:** Improvements in sexual function between the groups Data were analyzed using the Wilcoxon Signed-Rank Test for within-group comparisons and the Mann-Whitney U Test for between-group comparisons. Values are represented as mean ± standard deviation (SD). NS: not significant; * significant

Duration	Baseline	Day 30	Day 60	Day 90
Erectile Function				
Test	12.84 ± 1.76	*20.28 ± 2.72	*23.95 ± 3.79	*24.67 ± 4.52
Placebo	12.98 ± 2.11	*13.88 ± 1.80	*16.36 ± 3.04	*17.50 ± 4.55
P-value	1.000 (NS)	0.001	0.001	0.001
Orgasmic Function				
Test	4.14 ± 0.41	*6.79 ± 1.36	*7.93 ± 1.61	*8.21 ± 1.81
Placebo	4.40 ± 0.73	4.33 ± 0.79	*5.12 ± 1.50	*5.76 ± 2.07
P-value	0.2187 (NS)	0.001	0.001	0.001
Intercourse Satisfaction				
Test	6.77 ± 1.09	*10.02 ± 1.39	*11.67 ± 2.03	*12.26 ± 2.14
Placebo	6.90 ± 1.10	7.21 ± 1.00	*8.45 ± 1.81	*9.43 ± 2.46
P-value	0.453 (NS)	0.001	0.001	0.001
Overall Satisfaction				
Test	3.91 ± 0.75	*6.86 ± 1.17	*7.58 ± 1.47	*7.51 ± 2.16
Placebo	3.60 ± 0.70	*4.31 ± 0.75	*4.74 ± 1.15	*5.50 ± 1.33
P-value	0.126 (NS)	0.001	0.001	0.001

Assessment of changes in the mean sexual desire between the groups

**Figure 2:**
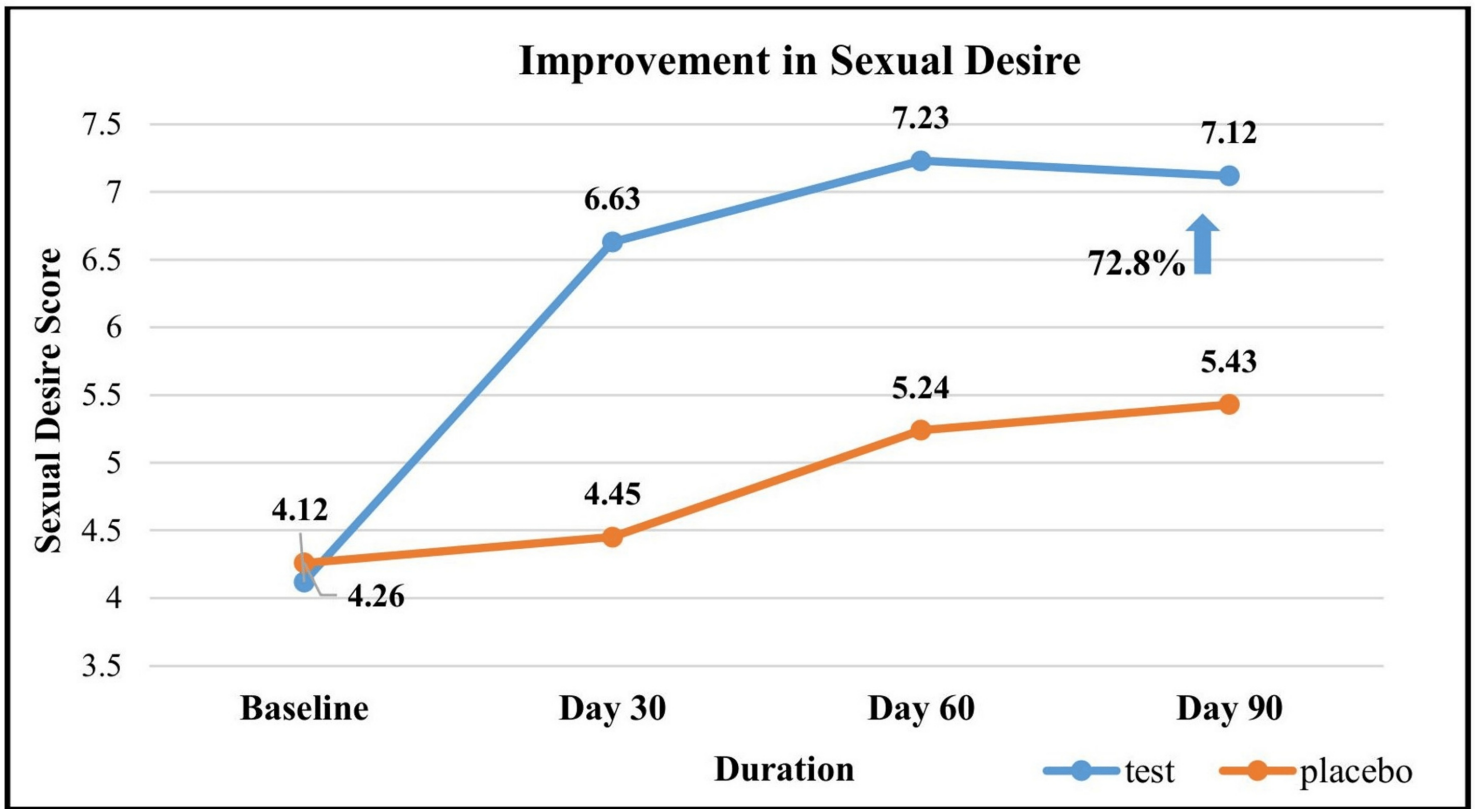
Assessment of changes in the mean sexual desire between the groups

At baseline, the mean sexual desire scores (IIEF) were similar in both groups. The test group showed a significant increase in sexual desire, with a 60.9% rise by day 30, 75.7% by day 60, and 72.8% by day 90. In contrast, the placebo group exhibited a non-significant increase of 4.5% by day 30, followed by a significant increase of 23% by day 60 and 27.5% by day 90. The test group demonstrated significantly greater improvement than the placebo group from day 30 onward (Figure 2).

Assessment of changes in EDITS scores

The test group showed significantly greater improvements in participant and partner satisfaction, with EDITS scores increasing from 40.26 to 47.37 for participants and from 18.49 to 22.00 for partners by day 90, compared to minimal changes in the placebo group (Table 4).

**Table 4:** Changes in mean EDITS scores between groups Data are presented as mean ± standard deviation (SD). Statistical significance was determined using the Mann-Whitney U Test, with * (p<0.001) indicating a significant difference. EDITS: Erectile Dysfunction Inventory of Treatment Satisfaction questionnaire

Duration	30 Days	60 Days	90 Days	P-value
Participant EDITS				
Test	40.26 ± 5.59	44.95 ± 3.73	47.37 ± 6.41	*0.001
Placebo	28.26 ± 4.97	30.81 ± 3.94	28.71 ± 5.99	*0.001
Partner EDITS				
Test	18.49 ± 2.45	20.40 ± 1.88	22.00 ± 3.14	*0.001
Placebo	12.57 ± 2.38	13.86 ± 1.98	13.45 ± 2.47	*0.001

Assessment of changes in the mean serum total testosterone between the groups

Serum total testosterone in the test group increased by 46.5%, from 338.25 at baseline to 495.51 on day 90, while the placebo group saw only a 3.3% increase, from 348.21 to 359.54. The test group showed a significantly greater change than the placebo group on day 90 (Table 5).

**Table 5:** Changes in the mean serum total testosterone between the groups Data were analyzed using the Wilcoxon Signed-Rank Test for within-group comparisons and the Mann-Whitney U Test for between-group comparisons. NS: not significant; * significant

Duration (Days)	Test (N = 43)	Placebo (N = 42)	P-value
Baseline	338.25 ± 160.16	348.21 ± 154.50	0.802 (NS)
Day 90	*495.51 ± 176.14	*359.54 ± 152.47	0.001

Assessment of changes in erection quality and sexual encounter profile

The QEQ consists of six questions scored from 1 to 5 and then converted to a 0-100 scale, with higher scores indicating better quality of erections, where Q1 ranges from "Almost always or always" (5) to "Almost never or never" (1), and Q2-Q6 range from "Very satisfactory" (5) to "Very unsatisfactory" (1). Whereas, the sexual encounter profile was assessed using a participant-reported daily diary card.

**Figure 3:**
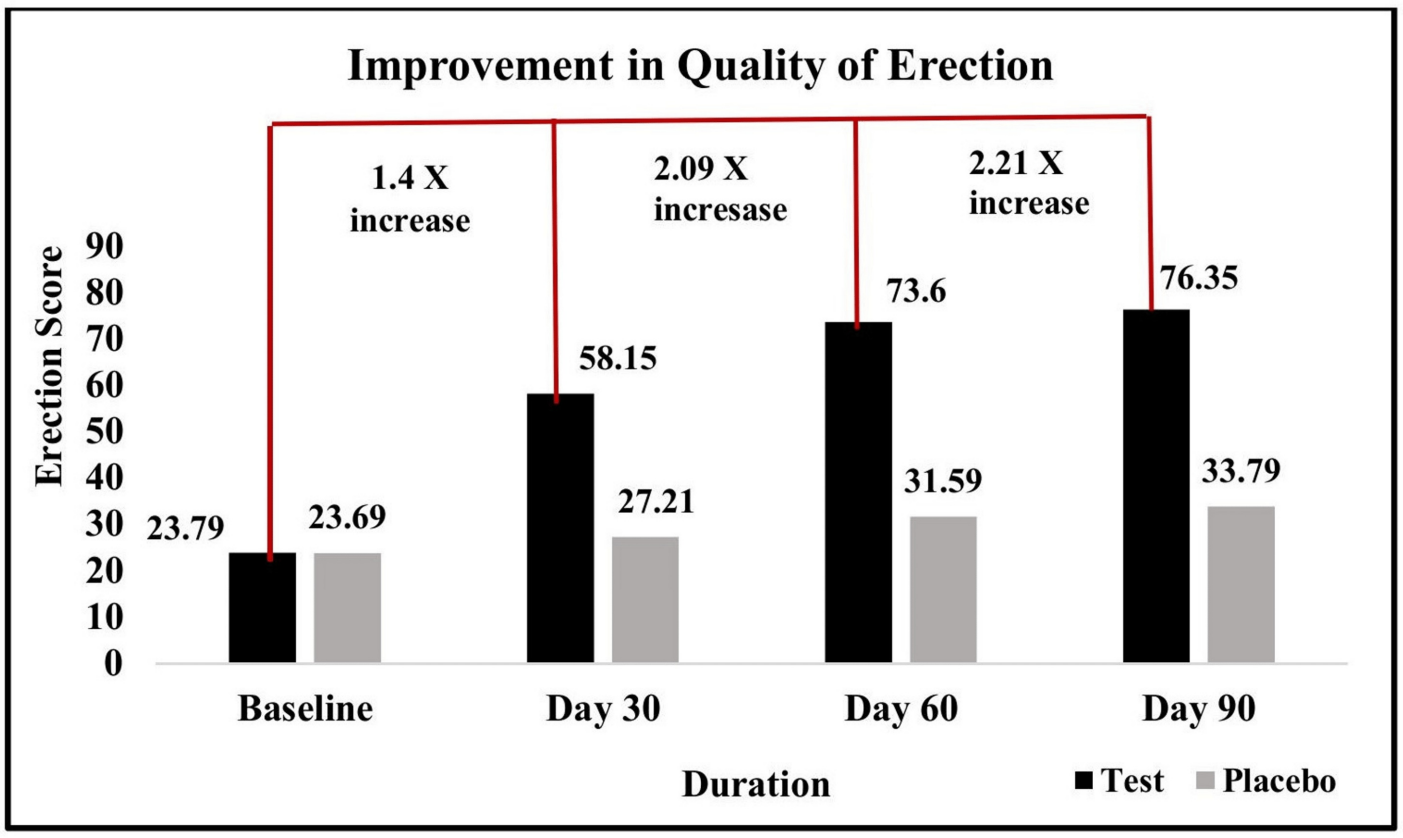
Changes in penile erection quality

**Figure 4:**
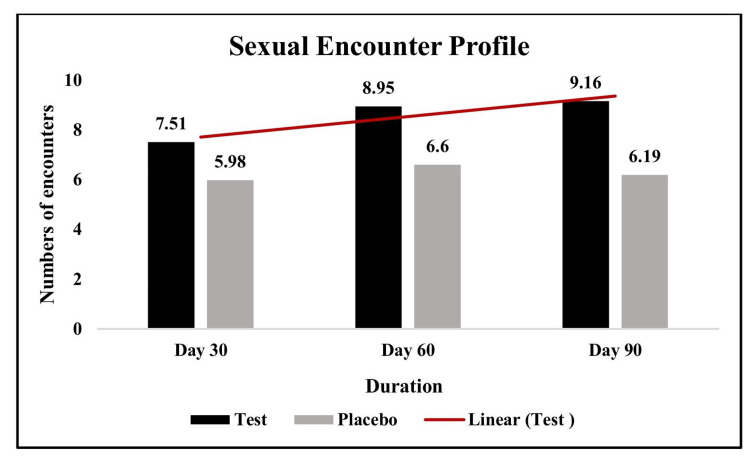
Changes in sexual encounter profile

The test group demonstrated significantly greater improvements in erection quality and frequency of sexual encounters compared to the placebo group. The mean quality of erection score in the test group increased from 23.79 at baseline to 76.35 by day 90, whereas the placebo group’s score rose modestly from 23.69 to 33.79. Similarly, the number of sexual encounters in the test group increased from 7.51 on day 30 to 9.16 by day 90, while the placebo group showed only a slight rise from 5.98 to 6.19 over the same period (Figures 3-4).

Assessment of laboratory and vital parameters

No significant changes were observed in hemogram, lipid profile, liver and renal function, or urine analysis in either group throughout the study. Vital parameters, including body temperature, pulse rate, and respiratory rate, showed no clinically relevant changes and remained within normal limits, with no statistically significant differences between the groups.

Assessment of drug compliance, tolerability, and AEs

Both groups demonstrated excellent drug compliance (≥97%) and good to excellent tolerability, as assessed by physicians, with no significant differences between groups. The observed AEs in the placebo group included burning throat, abdominal fullness, loose motion, and cough in five patients. In the test group, dyspepsia, body pain, headache, and mouth ulcers were reported in four patients. All AEs were mild in nature and resolved without the need for treatment discontinuation.

In-vitro PDE5 inhibitory activity

**Figure 5:**
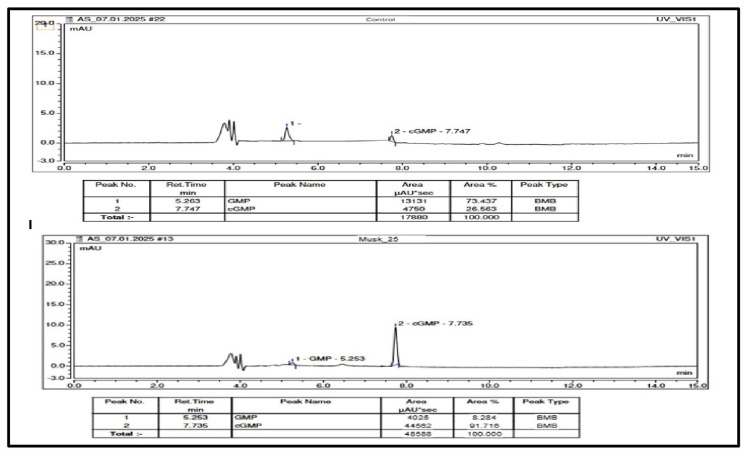
Chromatographic analysis of PDE5 inhibition assay

In-vitro studies were conducted to evaluate the polyherbal formulation's ability to inhibit PDE5 activity, a key target in ED. The assay showed a dose-dependent inhibition, with up to 99% inhibition at 0.5 mg/mL of solution of formulation. The PDE5 assay was performed according to a method adapted from Lin et al., with minor modifications. The inhibitory effect was quantified by measuring the remaining cGMP using HPLC, indicating the formulation's potential efficacy in treating ED (Figure 5) [22].

An investigational polyherbal formulation was tested at five concentrations to evaluate its potency and assess anti-PDE5 activity. The results demonstrate that the investigational product exhibits dose-dependent inhibition of PDE5, as shown in Table 6.

**Table 6:** Inhibition of PDE5 by the investigational formulation across varying concentrations

Concentration (mg/mL)	PDE5 Inhibition (%)
0.05	10
0.1	14
0.2	25
0.3	40
0.5	99.1

## Discussion

This study evaluated the efficacy and safety of test formulation tablets compared to placebo in participants with ED across four centers in India. Participants were randomized 1:1 into two groups: The test group (polyherbal formulation) and the placebo group (placebo), receiving two tablets twice daily after meals for 90 days.

In this study, the test group demonstrated significant efficacy in improving ED compared to placebo. The test group exhibited a 92.2% increase in mean EF, with 60.5% of participants reporting no ED, while the placebo group only had a 34.8% increase and 16.7% reported no dysfunction. Additionally, the test group showed substantial improvements in sexual desire (72.8%), orgasmic function (98.3%), and overall satisfaction (92.1%). Erection hardness scores and mean intra-vaginal ejaculation latency times were also significantly better in the test group. Moreover, 93% of participants in the test group reported substantial improvement as assessed by investigators, while 90.7% reported improvement according to self-assessment. Both groups achieved 100% drug compliance. The in-vitro study results also showed the polyherbal formulation inhibited PDE5 activity, with up to 99% inhibition at 0.5 mg/mL of concentration. This effect was quantified by measuring remaining cGMP using HPLC, supporting its potential efficacy in treating ED.

The superior efficacy of intervention polyherbal formulation tablets in enhancing ED compared to placebo is likely due to the synergistic effects of its ingredients, particularly *T. terrestris*, which is known for its aphrodisiac properties in managing ED [23].* T. terrestris* helps increase the release of nitric oxide from the endothelium and nitrergic nerve endings, relaxes the corpus cavernosum muscle, and increases intracavernous pressure, thus improving penile erection [24,25] *T. terrestris* also helps to increase dehydroepiandrosterone-sulfate (DHEA-S) levels and thereby improve sexual function in men [26].

It has been observed in the research study that Ashwagandha supplementation improves sexual function in male rats via activating the Nrf2/HO-1 pathway while inhibiting the NF-κB levels [27]. Ashwagandha helps in building muscle strength and also improves spermatogenesis [28,29]. Ashwagandha effectively improves an individual's resistance to stress and helps in relieving psychological elements involved in ED [30]. *Asparagus racemosus* is a well-known Ayurvedic rasayana that prevents aging, increases longevity, imparts immunity, improves mental function, and vigor, and adds vitality to the body [12]. It is extensively used in male genital dysfunctions, oligospermia, spermatogenic irregularities, and other male disorders [31,32]. *Mucuna pruriens* possess androgenic properties [33]. *M. pruriens* seed extract has a definite positive effect on male reproductive functions in terms of hormone profile, organ weights, semen quality, and quantity [34]. *M. pruriens* reduces stress and oxidative stress [35] and improves the quality of semen in infertile men [36]. *Pueraria tuberosa* possesses an androgenic effect and it helps increase sexual behavior [37]. Asparagus adscendus helps in improving fertility and vitality in women and men. It calms down nerve cells and prevents the risk of nervous disorders like depression, anxiety, and stress [38]. *Myristica fragrans* possesses aphrodisiac activity, increasing both libido and potency, which might be attributed to its nervous stimulating property [39,40]. Nutmeg is one of the best drugs used in delaying ejaculation time and thus sustaining the erection during intercourse [41]. Akarkarabha possesses aphrodisiac, antidepressant, androgenic and spermatogenetic properties [42].

The L-arginine-nitric oxide-cGMP pathway plays a key role in smooth muscle relaxation and penile erection, with PDE5 degrading cGMP to regulate this process. Inhibitors enhance cGMP levels by blocking PDE5. Our in-vitro study aligns with this mechanism, demonstrating that the polyherbal formulation effectively inhibits PDE5, potentially amplifying the cGMP pathway to improve EF [43].

Both test and placebo were well-tolerated, with mild AEs and no serious safety concerns. Both drugs showed excellent tolerability, with no significant changes in lab investigations or vital signs, indicating their safety. Therefore, the polyherbal formulation is deemed safe and effective for ED.

The test product is composed of a blend of herbal ingredients that target both physiological and psychological aspects of ED. Ingredients like *T. terrestris* and Ashwagandha not only improve the physical parameters of EF but also reduce stress, which is a common contributor to ED. The significant improvements in EF and associated parameters in the test group highlight the potential of polyherbal formulation as a potent treatment for ED. The high percentages of participants experiencing complete relief from ED further emphasize its effectiveness.

In summary, the test product demonstrated superior efficacy in improving ED and related sexual health outcomes compared to placebo, with a favorable safety profile. The synergistic effects of its herbal ingredients contribute to its potency, making it a promising therapeutic option for ED. However, further studies with larger sample sizes, and extended durations, are needed to confirm these findings and address the limitations of the current study.

While the study demonstrates promising results, certain limitations must be acknowledged. A larger sample size and extended follow-up periods are necessary to further validate the efficacy and safety of the polyherbal formulation in ED. Additionally, future research exploring its potential applications in other sexual disorders could broaden its therapeutic scope, offering greater clinical utility.

## Conclusions

The test group significantly enhanced various parameters of ED, including EF, sexual desire, orgasmic function, and overall satisfaction. Notably, participants receiving the test group exhibited superior treatment satisfaction compared to those on the placebo, along with a marked increase in serum total testosterone levels, and demonstrated a favorable safety profile. These findings substantiate the efficacy and safety of polyherbal formulation as a therapeutic option for mild to moderate ED. Thus, polyherbal formulation represents a promising intervention in the management of ED.
